# Neutrophil-to-lymphocyte ratio and its association with latent tuberculosis infection and all-cause mortality in the US adult population: a cohort study from NHANES 2011–2012

**DOI:** 10.3389/fnut.2024.1467824

**Published:** 2024-10-03

**Authors:** Yingxiu Huang, Ting Ao, Peng Zhen, Ming Hu

**Affiliations:** Department of Infectious Disease, Beijing Luhe Hospital, Capital Medical University, Beijing, China

**Keywords:** neutrophil to lymphocyte ratio, NLR, latent tuberculosis, LTBI, NHANES, mortality

## Abstract

**Background:**

There has been little study done on the possible connection between all-cause mortality and the neutrophil-to-lymphocyte ratio (NLR), particularly in individuals with latent tuberculosis infection (LTBI). The objective of this research was to examine the correlation between the NLR and LTBI, along with their effects on all-cause mortality in a cohort of individuals who had either LTBI or not.

**Methods:**

This research incorporated data from the National Health and Nutrition Examination Survey (NHANES) 2011–2012, with a total of 4938 subjects involved. To investigate the connection between LTBI and variables, multivariable logistic regression models were used. Multivariable Cox proportional hazards models and Kaplan-Meier (KM) survival curves were employed to examine the association between NLR and all-cause death in individuals with and without LTBI.

**Results:**

When analyzed as a continuous variable, The calculated odds ratios (ORs) for the different models-Model 1, Model 2, and Model 3 were 0.86, 0.83, and 0.84 (*P* < 0.005). NLR was evaluated as a categorical parameter, revealing that individuals in the tertile T3 had a notably lower rate of LTBI in comparison to those in the T1 group. After adjusting for different confounders, the odds ratio for T3 varied in the various models, being 0.75 (0.60∼0.95), 0.69 (0.54∼0.89), and 0.71 (0.56∼0.92), respectively. Additionally, higher NLR was significantly link to a greater risk of all-cause mortality in individuals with or without LTBI. Following multivariate adjustment, an 8% (Model 3, HR 1.08, 95% CI 1.05–1.12, *P* < 0.001) greater risk of mortality from all-cause was linked to every unit rise in NLR.

**Conclusion:**

Results from the study revealed a negative correlation between NLR and the likelihood of LTBI as well as a higher risk of death from all causes. Therefore, NLR may be a helpful technique for risk categorization in the adult LTBI in the United States. To clarify the underlying mechanisms and any therapeutic implications of these findings, more investigation is necessary.

## Introduction

Tuberculosis (TB), attributable to Mycobacterium tuberculosis (M.tb), represents a major worldwide infectious challenge, resulting in over 10 million cases and 1.5 million deaths annually (1). A distinctive feature of M.tb is its ability to persist latently within the host, leading to asymptomatic infection and the potential for reactivation. Individuals with asymptomatic M.tb infection are classified as having latent tuberculosis infection (LTBI), affecting approximately 25% of the global population (2). Those with LTBI face a 5–10% lifetime risk of progressing to active TB (3). Key factors, including compromised immune systems due to conditions like malignancy, HIV infection, malnutrition, diabetes, or smoking, amplify the risk of developing active TB among those with LTBI (4). The majority of active TB cases stem from the reactivation of LTBI, highlighting the pivotal role of LTBI in TB epidemiology. Consequently, addressing LTBI through prevention and treatment is crucial for reducing TB-related morbidity, mortality, and further transmission (4).

The neutrophil-to-lymphocyte ratio (NLR), a simple hematological parameter derived from a complete blood count, has been identified as a new inflammatory marker that increases in various conditions, including malignancies (5), non-alcoholic fatty liver disease risk (6), and cerebrovascular diseases (7). Studies have shown that NLR is more effective than C-reactive protein (CRP), leukocyte count, and lymphocyte count in determining bacteremia in infectious diseases (8). In previous studies, NLR has been discovered to be a helpful biomarker for predicting prognosis or severity in different disease, such as sepsis (9), bacterial meningitis (10), military tuberculosis (11), and tuberculosis meningitis (12). Previous studies have linked NLR to tuberculosis (TB) infection. Notably, Yoon et al. (13) found that NLR levels in individuals with pulmonary TB were significantly lower than those with bacterial community-acquired pneumonia. Jeon’s (14) research also indicated lower NLR in TB patients versus those with other respiratory infections. Similarly, Liu et al. (15) reported reduced NLR in spinal TB compared to pyogenic spinal infections. Cursi’s (16) study showed higher mean NLR in pediatric TB patients and in those with active TB.

In the US population, there was no known correlation between all-cause mortality and NLR and LTBI. For this reason, this study uses a thorough population-based survey “to evaluate the connection between NLR, LTBI, and all-cause mortality, providing important information on the health of individuals in the US.

## Materials and methods data source

### Study design and participants

The National Center for Health Statistics (NCHS) of the Centers for Disease Control and Prevention (CDC) runs the NHANES database, which provided the data for this study (17). Data on the overall health and dietary status of civilian, non-institutionalized Americans are gathered by the nationally representative NHANES survey. (18). Before taking part in the survey, each participant gave their informed consent, and there is no individually identifiable patient data in the NHANES dataset.

**FIGURE 1:**
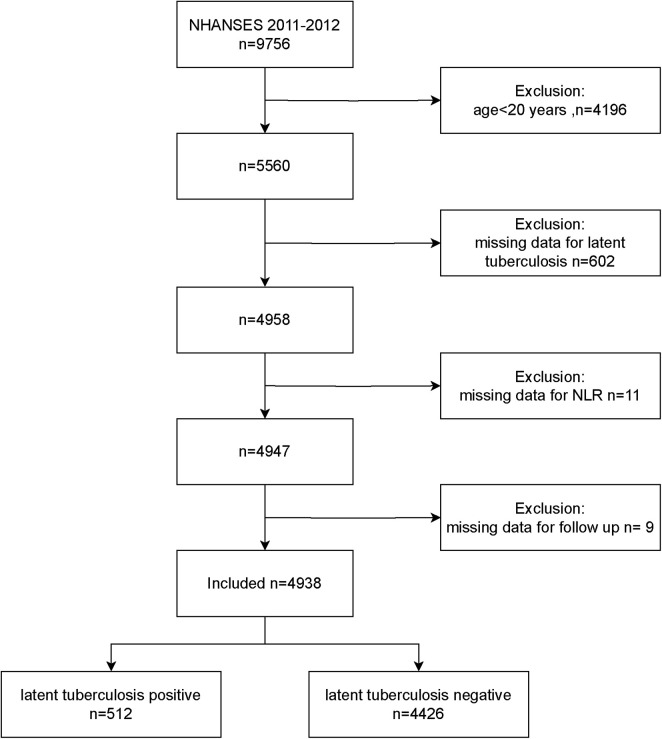
Flowchart of study.

5,560 adults of 9,756 participants were included in the study during the course of the NHANES 2011–2012 cycle. Once those without NLR data or LTBI status were eliminated, along with those who were missing follow-up. The remainder of 4,938 provided the basis for the analysis (Figure 1).

### NLR measurement

Blood was collected at the Mobile Examination Center (MEC), and CBC tests, including lymphocyte and segmented neutrophil counts, were performed using the Beckman Coulter^®^ HMX on the participant’s EDTA-anticoagulated blood samples. The results were expressed as × 103 cells/μL for neutrophil and lymphocyte counts. The calculation of NLR involved dividing the total number of neutrophils by the total number of lymphocytes (NLR = segmented neutrophil counts/lymphocyte counts) (19).

### LTBI measurement

The QFT-GIT, or QuantiFERON^®^-TB Gold In Tube system involves venipuncture-based whole blood collection using specialized tubes: Nil control, TB Antigen, and Mitogen (positive control). In the NHANES MEC, on the same day that each eligible participant underwent a TB skin test, a blood test was also administered to screen for tuberculosis infection. For this procedure, 1 milliliter of blood was collected from each subject via venipuncture and directly transferred into three QFT GIT blood collection tubes in the following sequence: Nil (grey cap), TB Antigen (red cap), and Mitogen (purple cap). After incubation at 37°C + 1°C for 16 to 24 hours, plasma is extracted. Interferon-gamma (IFN-γ) response to TB Antigen is measured using enzyme-linked immunosorbent assay (ELISA) (20, 21). A positive result indicates significantly higher IFN-γ response to TB Antigen compared to Nil control. Positive QFT-GIT results were determined using NHANES criteria: 1) a nil value ≤ 8.0 IU/mL of gamma interferon (IF), 2) a TB antigen value minus the nil value ≥ 0.35 IU/mL of gamma interferon, and 3) the TB antigen value minus the nil value ≥ 25% of the nil value. Participants with incomplete or indeterminate QFT-GIT results were not included. An indeterminate result is indicated by a mitogen response < 0.5 IU/mL and a concurrent negative response to TB antigens. Positive results categorize participants as having latent tuberculosis infection (LTBI), while negative results classify them as non-LTBI. Indeterminate participants are considered missing data in the analysis. (22, 23).

### Assessment of all-cause mortality and follow-up

By integrating data from the National Death Index, available at https://www.cdc.gov/nchs/data-linkage/mortality-public.htm, with NHANES data, mortality status was ascertained. Data from the NDI was used to classify participants as either living or dead. The follow-up period was determined by subtracting the NHANES examination date from the death date or December 31, 2019, whichever came first. To identify the underlying causes of mortality, the International Classification of Diseases, 10th Revision (ICD-10) was used. A follow-up period of 95 months was the median (interquartile range: 90–102).

### Covariates

The study considered several potential confounding factors by referencing prior research and utilizing clinical judgment. Included in these considerations were age, gender, family poverty to income ratio (PIR), body mass index (BMI), educational level, marriage statue, smoking status, alcohol intake, diabetes, hypertension, hemoglobin, and platelet count. Age was considered a continuous factor, whereas gender was classified as male and female. Marital status was classified as either being married or living with a partner, or living alone. Educational level was classified into three groups: less than 9 years, 9 to 12 years, and more than 12 years. Smoking status was classified as smoking or non-smoking. Likewise, alcohol use was categorized into non-drinkers and drinkers. Diabetes diagnosis in the study was determined using the Diabetes Questionnaire (DIQ), specifically question DIQ010, which if participants had ever been diagnosed with diabetes by a health professional. Individuals who responded yes to the DIQ010 were classified as having diabetes (24). In this research, hypertension was identified using question BPQ020 from the Blood Pressure & Cholesterol Questionnaire (BPQ), which asked participants if they had been diagnosed with hypertension by a healthcare professional. Participants responding affirmatively to the BPQ020 question were categorized as having hypertension (7). The assumption for coronary heart disease (CHD) was the affirmative response on MCQ 160c: You’ve been diagnosed with coronary heart disease (7).

### Statistical analysis

Participants with and without LTBI compared and aggregated the participant baseline characteristics. The research divided the data into two types: continuous and categorical variables. Continuous variables were further classified according to whether their distribution was normal or not. The Student’s t-test was utilized for comparing continuous variables that followed a normal distribution, and were presented as mean with standard deviation. Variables that did not follow a normal distribution were presented as median with interquartile range (IQR) and compared using the Wilcoxon rank-sum test. Categorical variables, presented as percentages, were compared using the chi-squared test. The significance of differences among groups categorized by tertiles of NLR was assessed using either the Kruskal-Wallis test or one-way analysis of variance.

Logistic regression was utilized to determine the OR with a 95% CI regarding the association between NLR and LTBI. To investigate the relationship between NLR and mortality from all-cause, we employed cox hazards models to compute HRs with 95% CIs. To minimize the risk of overadjustment for variables that would influence the association between NLR and mortality from all-cause, we employed three different models. NLR was stratified into tertiles and analyzed by Cox proportional hazards models, with tertile T1 as the reference group. The Kaplan-Meier method was utilized to estimate the event-free survival rates among the groups, and the log-rank test was employed to compare them. For data with a missing rate of less than 30%, we use K nearest neighbor (KNN) algorithm (25).

Data analysis was conducted with R software version 4.1.1, the R survey package version 4.1.1, and Free Statistics software version 1.9.2 from R Foundation for Statistical Computing in Vienna, Austria (26). Statistical significance was determined with a two-tailed p-value less than 0.05. This cross-sectional study was reported in accordance with the guidelines outlined in the Strengthening the Reporting of Observational Studies in Epidemiology (STROBE) statement (27).

## Results

### Participants demographics at baseline

The study population’s characteristics are presented in Table 1. For the purposes of this study, a total of 4,938 individuals qualified. Among them, 512 individuals were tested positive for LTBI based on QFT-GIT test. 49.3% of the included population was male. The average age was 48.7 years. The LTBI group was older, had lower levels of education, lower income, higher BMI, higher rates of hypertension and diabetes, and neutrophil counts and NLR were lower in comparison to the non-LTBI group.

**TABLE 1:** Baseline characteristic of participants.

Characters	Total (*n* = 4938)	Non-LTBI (*n* = 4426)	LTBI (*n* = 512)	*P*
Gender, *n* (%)				<0.001
Male	2433 (49.3)	2139 (48.3)	294 (57.4)	
Female	2505 (50.7)	2287 (51.7)	218 (42.6)	
Age, years	48.7 ± 17.7	47.8 ± 17.8	56.2 ± 15.3	<0.001
PIR	2.4 ± 1.7	2.5 ± 1.7	2.2 ± 1.6	0.002
BMI, kg/m^2^	28.8 ± 6.9	28.9 ± 6.9	28.6 ± 6.7	0.398
Educational level, years, *n* (%)				<0.001
<9	463 (9.4)	353 (8)	110 (21.5)	
9–12	1710 (34.6)	1524 (34.4)	186 (36.3)	
> 12	2763 (56.0)	2547 (57.6)	216 (42.2)	
Race, n (%)				<0.001
Non-Hispanic White	489 (9.9)	413 (9.3)	76 (14.8)	
Non-Hispanic Black	1848 (37.4)	1776 (40.1)	72 (14.1)	
Mexican American	1265 (25.6)	1142 (25.8)	123 (24)	
Other	1336 (27.1)	1095 (24.7)	241 (47.1)	
Marriage statue, *n* (%)				0.007
Living with partner	2792 (56.6)	2474 (55.9)	318 (62.1)	
Living alone	2144 (43.4)	1950 (44.1)	194 (37.9)	
Diabetes, *n* (%)				<0.001
No	4312 (87.3)	3896 (88)	416 (81.2)	
Yes	626 (12.7)	530 (12)	96 (18.8)	
Hypertension.cat, *n* (%)				0.004
No	3176 (64.3)	2876 (65)	300 (58.6)	
Yes	1762 (35.7)	1550 (35)	212 (41.4)	
Coronary heart disease, *n* (%)				0.227
No	4767 (96.5)	4268 (96.4)	499 (97.5)	
Yes	171 (‘3.5)	158 (3.6)	13 (2.5)	
Smoke, *n* (%)				0.025
No	2823 (57.2)	2554 (57.7)	269 (52.5)	
Yes	2115 (42.8)	1872 (42.3)	243 (47.5)	
Alcohol use, n (%)				<0.001
No	1173 (26.6)	1021 (25.7)	152 (34.5)	
Yes	3244 (73.4)	2955 (74.3)	289 (65.5)	
NLR	2.2 ± 1.2	2.2 ± 1.3	2.0 ± 1.0	0.002
lymphocyte (10^3^ cells/μL)	2.1 ± 1.1	2.1 ± 1.1	2.1 ± 0.6	0.858
Neutrophil (10^3^ cells/μL)	4.1 ± 1.7	4.1 ± 1.7	3.9 ± 1.5	0.004
Hemoglobin (g/dL)	13.9 ± 1.5	13.9 ± 1.6	13.8 ± 1.4	0.595
Platelet (× 10^9^/L)	236.4 ± 60.5	237.2 ± 60.4	229.4 ± 61.0	0.006

### Associations between NLR and LTBI

Using multivariate logistic regression, the research examined the correlation between NLR and LTBI by 3 different models. Three models were following: Model 1 was unadjusted; Model 2 was adjusted for gender, age, and race; and Model 3 included additional adjustments for educational level, BMI, PIR, alcohol consumption, smoking status, diabetes, hypertension, and CHD. The results in Table 2 show that an increase in NLR was associated with a lower risk of LTBI. When analyzed as a continuous variable, the ORs for Models 1, 2, and 3 were as follows: 0.86, 0.83, and 0.84 (*P* < 0.005). NLR was evaluated as a categorical measure, showing that subjects in tertile T3 exhibited a significantly lower incidence of LTBI compared to individuals in the T1 tertile. After adjusting for different confounders, the OR for T3 varied in the various models, being 0.75 (0.60∼0.95), 0.69 (0.54∼0.89), and 0.71 (0.56∼0.92), respectively. The trend test revealed a significant trend (*p* < 0.05) (Table 2).

**TABLE 2:** Associations between NLR and LTBI.

Variable	n.event_%	Model 1		Model 2		Model 3	
		OR (95%CI)	*P*-value	OR (95%CI)	*P*-value	OR (95%CI)	*P*-value
NLR	512 (10.4)	0.86 (0.78∼0.94)	0.002	0.83 (0.75∼0.92)	<0.001	0.84 (0.76∼0.93)	0.001
**NLR tertile**							
T1 (<1.61)	189 (11.5)	1 (Ref)		1 (Ref)		1 (Ref)	
T2(1.61–2.36)	177 (10.7)	0.93 (0.75∼1.15)	0.494	0.93 (0.74∼1.17)	0.546	0.94 (0.74∼1.18)	0.576
T3(> 2.36)	146 (8.9)	0.75 (0.6∼0.95)	0.015	0.69 (0.54∼0.89)	0.003	0.71 (0.56∼0.92)	0.008
P for trend			0.015		0.004		0.009

Model 1: unadjusted. Model 2: adjusted for gender, age, race. Model 3: adjusted for gender, age, race, education, BMI, PIR, alcohol, smoke, diabetes, hypertension, coronary heart disease. OR, odds ratio; CI, confidence interval.

### Associations between NLR and all-cause mortality in participants with LTBI and non-LTBI

**FIGURE 2:**
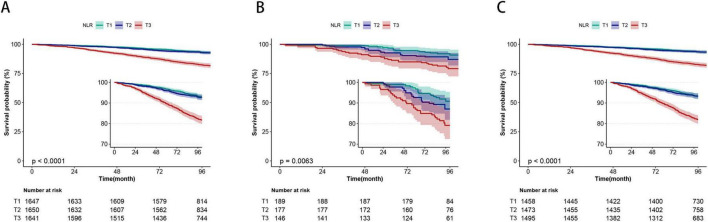
Kaplan-Meier survival curve for all-cause of all included participants **(A)**, with LTBI **(B)**, and non-LTBI **(C)**.

With a median follow-up of 95 months (IQR: 90–102months), 518 (10.5%) of the 4938 participants died. For the total population, in Model 1 with unadjusted, rising NLR values were significantly associated with an increased risk of mortality from all-cause (HR 1.16, 95% CI: 1.14–1.19, *P* < 0.001) (see Table 3). Following multivariate adjustment, per one-unit increase in NLR was associated with a 7% increased risk of all-cause mortality in Model 2 (HR 1.07, 95% CI: 1.04–1.10, *P* < 0.001) and an 8% increased risk in Model 3 (HR 1.08, 95% CI: 1.05–1.12, *P* < 0.001) (see Table 3). Cox regression modeling indicated a marked rise in death from all-cause for the T3 group, with hazard ratios of 2.82 (95% CI: 2.27–3.52, *P* < 0.001) in Model 1, 1.89 (95% CI: 1.50–2.39, *P* < 0.001) in Model 2, and 1.80 (95% CI: 1.42–2.27, *P* < 0.001) in Model 3, relative to the lower NLR T1 group (see Table 3). Survival curve analysis revealed a significant reduction in survival rates for the higher T3 group compared to the T1group (*P* < 0.0001) (see Figure 2A).

**TABLE 3:** Associations of NLR with all-cause mortality in participants with LTBI and non-LTBI.

Variable	n.event_%	Model 1		Model 2		Model 3	
		HR (95%CI)	*P*-value	HR (95%CI)	*P*-value	HR (95%CI)	*P*-value
NLR	518 (10.5)	1.16 (1.14∼1.19)	<0.001	1.07 (1.04∼1.1)	<0.001	1.08 (1.05∼1.12)	<0.001
**NRL tertile**							
T1 (<1.61)	110 (6.7)	1(Ref)		1(Ref)		1(Ref)	
T2 (1.61–2.36)	117 (7.1)	1.06 (0.82∼1.38)	0.637	0.99 (0.76∼1.28)	0.914	0.98 (0.75∼1.25)	0.856
T3(> 2.36)	291 (17.7)	2.82 (2.27∼3.52)	<0.001	1.89 (1.5∼2.39)	<0.001	1.80 (1.42∼2.27)	<0.001
**LTBI**							
T1 (<1.61)	16 (8.5)	1(Ref)		1(Ref)		1(Ref)	
T2 (1.61–2.36)	21 (11.9)	1.44 (0.75∼2.77)	0.268	1.43 (0.74∼2.76)	0.292	1.60 (0.82∼3.11)	0.17
T3(> 2.36)	29 (19.9)	2.53 (1.37∼4.65)	0.003	2.24 (1.18∼4.25)	0.013	2.21 (1.15∼4.25)	0.018
**Non-LTBI**							
T1 (<1.61)	94 (6.4)	1(Ref)		1(Ref)		1(Ref)	
T2 (1.61–2.36)	96 (6.5)	1.01 (0.76∼1.34)	0.938	0.92 (0.69∼1.23)	0.576	0.89 (0.66∼1.18)	0.428
T3 (> 2.36)	262 (17.5)	2.89 (2.28∼3.66)	<0.001	1.85 (1.44∼2.37)	<0.001	1.73 (1.35∼2.22)	<0.001

Model 1: unadjusted. Model 2: adjusted for gender, age, race. Model 3: adjusted for gender, age, race, education, BMI, PIR, alcohol, smoke, diabetes, hypertension, coronary heart disease. HR, hazard ratio; CI, confidence interval.

Among 512 participants with LTBI, 66 (12.9%) died. In the participants with LTBI, greater NLR T3 group exhibited increased all-cause mortality (HR: 2.21, 95% CI: 1.15–4.25, *P* = 0.018, in model 3) compared to the T1 group (see Table 3). Kaplan-Meier survival analysis revealed a notable decrease in survival rates for the T3 group relative to the T1 group (*P* = 0.0063) (see Figure 2B). For participants without LTBI, the result was similar, the T3 group demonstrated a significantly elevated risk of all-cause mortality (HR: 1.73, 95% CI: 1.35–2.22, *P* < 0.001) relative to the T1 group (see Table 3). KM curve analysis result showed T3 group had lower survival rate compared to T1 group (*P* < 0.0001) (see Figure 2C).

### ROC analysis

We conducted an area under the receiver operating characteristic (ROC) curve analysis, and found that the optimal NLR cutoffs of NLR for all-cause mortality was 2.38 (specificity:0.70, sensitivity: 0.56).

### Subgroup analysis

**FIGURE 3:**
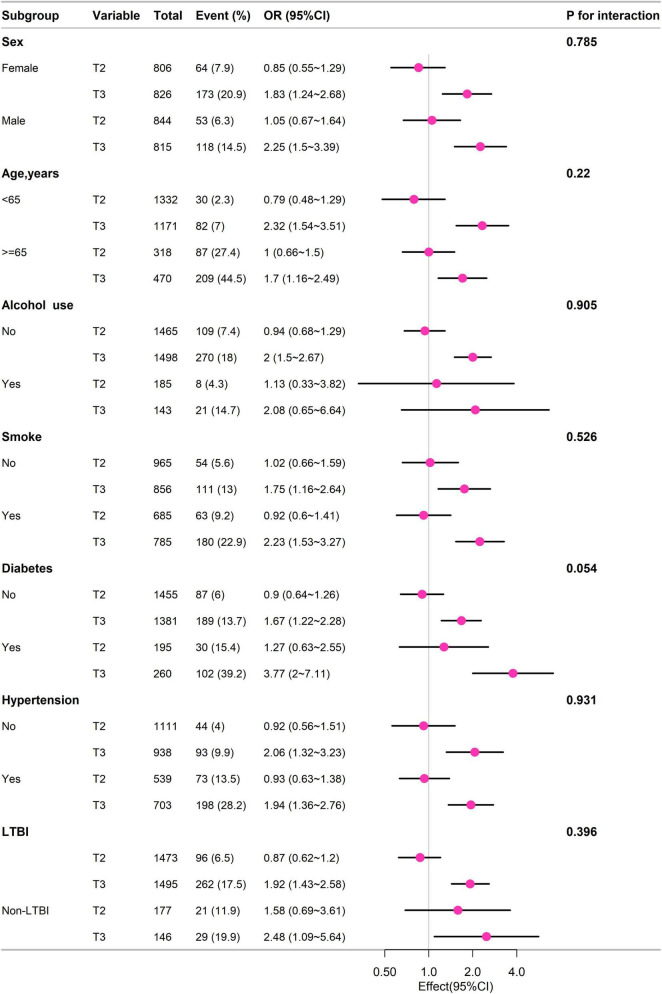
Subgroup analysis of different groups based on sex, age (< 65 years, ≥ 65 years), alcohol use, smoke, diabetes, hypertension, LTBI, and non-LTBI, adjustment for gender, age, race, education, BMI, PIR, alcohol, smoke, diabetes, hypertension, coronary heart disease.

Subgroup analysis of different groups based on sex, age (< 65 years, ≥ 65 years), alcohol use, smoke, diabetes, hypertension, LTBI, and non-LTBI, adjustment for gender, age, race, education, BMI, PIR, alcohol, smoke, diabetes, hypertension, coronary heart disease. The results remained stable between subgroups (*p* for interaction > 0.05) (Figure 3).

## Discussion

The current study revealed that NLR showed a negative correlation with LTBI, and NLR exhibited a positive relationship with mortality from all-cause. To the extent of our understanding, this is the first research to explore the relationship between NLR, LTBI, and death risk among the US adult population.

Because of their affordability and easy application, blood tests frequently assess the levels of neutrophils and lymphocytes. When considered collectively, these counts provide crucial insights into the equilibrium between adaptive immunity, represented by lymphocytes, and innate immunity, represented by neutrophils, as well as the overall state of systemic inflammation. Studies have revealed that NLR is a more robust predictor than either measure considered individually. Earlier research has shown the association with NLR and tuberculosis infection. Yoon et al. (13) reported that the levels of NLR in the serum were notably reduced among individuals diagnosed with pulmonary tuberculosis compared to those with bacterial community-acquired pneumonia (3.67 ± 2.12 vs. 14.64 ± 9.72, *P* < 0.001). Jeon’s (14) study also found that the NLR was notably lower in TB patients compared to those with other infectious lung diseases. Liu et al. (15) observed that the NLR was notably reduced in patients with spinal tuberculosis in contrast to those with pyogenic spinal infections (*P* < 0.001). While Cursi’s (16) study revealed that the mean NLR values were higher in pediatric patients with TB compared to those without TB (2.04 (± 2.04) vs. 1.21 (± 1.18), *P* < 0.001) and in pediatric patients with active TB compared to those with non-active TB (2.43 (± 2.30) vs. 1.32 (± 1.13), *P* = 0.001). Wang et al. (28) discovered that in patients with LTBI in end-stage renal disease, elevated NLR was a significantly protective factor in final multivariate logistic regression analyses (aOR: 0.50, 95% CI 0.28–0.89, *P* = 0.02). Our study results demonstrated that the NLR was lower in participants with LTBI compared to those with non-LTBI in US adults, and there was a negative association between NLR and LTBI. Our findings suggested that NLR was a reliable marker of inflammation. Our results align with and reinforce the existing body of knowledge on NLR.

Numerous prior investigations have confirmed that NLR correlates with adverse outcomes in various diseases, including sepsis (29), COVID-19 (30), COPD (31), cancer (32, 33), hypertension (7), and heart failure (34). Studies have also explored the association between NLR and TB prognosis. Gu et al. (12) discovered that in tuberculosis meningitis patients without HIV infection from a Chinese hospital, the NLR serves as an independent risk factor for 28-day mortality (OR = 1.065, 95% CI = 1.001–1.133, *P* = 0.045). Han and colleagues (11) discovered that the NLR served as a significant predictor of in-hospital mortality (aHR: 1.08, 95% CI: 1.03–1.13) and 1-year mortality (aHR: 1.08, 95% CI: 1.05–1.12) in their study of 96 patients with miliary tuberculosis in South Korea. In individuals with LTBI, we discovered a positive relationship between NLR and mortality. Our outcome was in line with the ones mentioned above. The reason behind the correlation between the rise in NLR and poor outcomes has not been fully understood or explained yet. According to some research, comparatively low lymphocyte numbers in cancer patients may be linked to an inadequate response to chemotherapy concerning cell-mediated immunity (35, 36). The immunological response to Mycobacterium TB relies heavily on cell-mediated immunity, and an elevated NLR may suggest relative lymphopenia. Thus, the clinical progression of LTBI may be influenced by a similar mechanism.

Our research possesses several notable advantages. Firstly, it is the first research to evaluate the relationship between NLR and LTBI in adults living in the US with a large sample size. Second, the research categorized NLR into distinct variables, which helps mitigate confounding factors and strengthens the reliability of the findings.

However, the study had certain limitations. Firstly, there is a chance that some confounding variables such as chronic liver disease, renal disease, cancer, that might affect the association between NLR and mortality were not included in the analysis of adjusted confounding factors. Secondly, because this study’s data came from participants in the United States, more research is necessary to verify whether the findings can be applied to populations with LTBI abroad. Third, as judging NLR purely on basis of a single laboratory test may not be indicative of patients’ immunity. We need further research to establish a more comprehensive understanding of the change of NLR in disease diagnosis and prognosis, especially in the context of LTBI. Future research endeavors should incorporate longitudinal studies featuring interventional elements to elucidate the causal nexus between NLR and mortality.

## Conclusion

The study’s findings showed a negative correlation between NLR and the likelihood of LTBI as well as a higher risk of death from all-cause. Therefore, NLR may be a helpful technique for risk categorization in the adult LTBI in the United States. To clarify the underlying mechanisms and any therapeutic implications of these findings, more investigation is necessary.

## Data Availability

The raw data supporting the conclusions of this article will be made available by the authors, without undue reservation.
